# Perceptions of Factors Associated With Inclusive Work and Learning Environments in Health Care Organizations: A Qualitative Narrative Analysis

**DOI:** 10.1001/jamanetworkopen.2018.1003

**Published:** 2018-08-03

**Authors:** Jaya Aysola, Frances K. Barg, Ana Bonilla Martinez, Matthew Kearney, Kareha Agesa, Carlos Carmona, Eve Higginbotham

**Affiliations:** 1 Office of Inclusion and Diversity, Perelman School of Medicine, University of Pennsylvania, Philadelphia; 2 Leonard Davis Institute of Health Economics, University of Pennsylvania, Philadelphia; 3 Department of Medicine, Perelman School of Medicine, University of Pennsylvania, Philadelphia; 4 Department of Family Medicine and Community Health, Perelman School of Medicine, University of Pennsylvania, Philadelphia; 5 Center for Clinical Epidemiology and Biostatistics, Perelman School of Medicine, University of Pennsylvania, Philadelphia

## Abstract

**Importance:**

Diversifying the health care workforce remains a critical goal for health care organizations focused on reducing disparities in care. However, it remains unknown what factors create inclusive health system environments and help organizations retain a diverse workforce.

**Objective:**

To understand from members of the health care workforce what factors contribute to inclusive work and learning environments and what can be done to improve inclusion within health care organizations.

**Design, Setting, and Participants:**

A qualitative narrative analysis of responses to a weekly email call for narratives within health care organizations sent June 1, 8, 15, and 22, 2016. The email contained an anonymous link to 2 open-ended stimulus questions asking for stories reflecting inclusion or lack thereof within participants’ work environments as well as demographic questions. The study took place at 6 hospitals, including a free-standing children’s hospital and a Veterans Affairs medical center, 4 health sciences schools (Medicine, Nursing, Dental, and Social Policy and Practice), and outpatient facilities within a university-based health care system in Pennsylvania. There were 315 completed narratives submitted from health care system executives (n = 3), staff (n = 113), academic faculty (n = 97), trainees or students (n = 99), and 3 who declined to specify their positions.

**Main Outcomes and Measures:**

Workplace experiences with inclusivity, implications of these experiences, and recommendations to improve inclusion within environments.

**Results:**

Of 315 narratives submitted from members of the health care system, in 188 (59.7%) the writer self-identified as female; in 10 (3.2%), as transgender/queer; in 38 (12.1%), as non-Hispanic black; in 152 (48.3%), as non-Christian; in 31 (9.8%), as having a language other than English as their primary language; and in 14 (4.4%), as having a disability. Analysis of the narratives revealed 6 broad factors that affected inclusion within health care organizations: (1) the presence of discrimination; (2) the silent witness; (3) the interplay of hierarchy, recognition, and civility; (4) the effectiveness of organizational leadership and mentors; (5) support for work-life balance; and (6) perceptions of exclusion from inclusion efforts. Challenges with inclusion had negative effects on job performance and well-being, with reports of stress, anxiety, and feelings of hopelessness. Most respondents referenced a systemic culture that influenced their interpersonal dynamics and provided specific strategies to improve organizational culture that focused on leadership training and expanding collegial networks.

**Conclusions and Relevance:**

This narrative analysis provides a taxonomy of factors that health care organizations can use to assess inclusion within their learning and work environments as well as strategies to improve inclusion and retain a diverse health care workforce.

## Key Points

**Question:**

What factors matter for creating inclusive clinical and learning environments within health care organizations?

**Findings:**

This qualitative analysis identifies 6 modifiable contributors to an inclusive culture and the implications of these factors on the well-being and performance of members of the health care workforce.

**Meaning:**

Understanding these factors provides health care systems a way to improve inclusivity and facilitate the retention of a diverse health professional student body and health care workforce to ultimately improve care delivery.

## Teaser

This qualitative analysis of health care student and professional responses to questions about inclusion and respect in respondents’ hospitals and health schools describes factors influencing their perceptions and strategies to improve inclusion and retain a diverse health care workforce.

## Introduction

Diversifying the health care workforce remains a critical goal for many health care organizations focused on improving clinician education and reducing disparities in access and care.^1,2,3,4,5,6,7^ Populations with equal access to care experience disparities in treatment despite well-intentioned efforts because of the structural bias ingrained in our health care systems and health care professionals’ implicit biases.^8,9,10,11^ Prior evidence demonstrates that the engagement of a diverse workforce reduces such biases and improves the cultural competencies of nonminority and minority health care professionals alike.^1,8,12^ Enhancing diversity brings together distinct minds with varying perspectives, backgrounds, and experiences, improving the way we generate medical knowledge and deliver care.^8,13,14,15^ Prior solutions for a diverse workforce have centered on recruitment and not retention,^2,16^ despite evidence of greater attrition among women and minorities.^17,18,19,20^ Organizational efforts that focus on creating an inclusive environment may promote greater retention of a diverse workforce, reduce the costs related to attrition, and ultimately affect patient satisfaction and care quality.^21^ However, there is a paucity of research on how health care organizations create a culture that promotes inclusive environments to achieve these goals. Prior work has conceptualized inclusion as a set of social processes that influence an individual’s access to information, sense of belonging and job security, and social support system.^22^ Health organizations lack sufficient understanding of the operational definition of inclusion to guide their efforts to improve culture. To address this gap, we conducted a narrative analysis to understand from members of the health care workforce what factors affect perceptions of inclusion in a health care system.

## Methods

### Data Collection

To conduct this narrative analysis, we used a culturally diverse study team that included 3 student researchers, a research coordinator, and 3 faculty members. We solicited anonymous narratives from employees, faculty, and students about their experiences with inclusion at 6 hospitals, including a free-standing children’s hospital and a Veterans Affairs medical center, and 4 health schools (Medicine, Nursing, Dental, and Social Policy and Practice) within a university-based health system. Administrators sent a weekly email call for narratives on June 1, 8, 15, and 22, 2016, to listservs representing different constituents within and across organizations affiliated with the academic health system. This email contained an anonymous link to an inquiry using REDCap,^23^ consisting of demographic questions and 2 open-ended stimulus questions designed to evaluate participant interpretations of inclusion:

1. Think about a time when you witnessed or participated in a situation where you or a colleague/member of [this organization] was treated in a manner that made you/them feel either included, valued, and welcome OR excluded, devalued, and unwelcome as a member of this [organization].
2. Please comment on your perception of the general climate at the [organization] with regards to inclusion and respect.

Respondents were instructed to avoid use of personal identifiers and were promised anonymity. We flagged and redacted any narratives with identifiable information. Some initiated a narrative but did not submit (n = 1270), and others submitted incomplete narratives (n = 47). All data from the responses were stored in REDCap with access limited to the study team. The University of Pennsylvania Institutional Review Board approved our study protocol and the recruitment language that detailed public use of only deidentified narratives. Participants consented for public use of their anonymized data by submitting a response, as indicated in the instructions given in the email. We followed Standards for Reporting Qualitative Research (SRQR) reporting guidelines in reporting this study.

### Data Analysis

We analyzed data from all submitted narratives with completed responses to both open-ended questions (n = 315). We evaluated the narratives with a focus on both structure and content, using the Labov and Waletzky model for narrative analysis.^24^ We coded core features of each narrative (introduction, presenting problem, complicating factors, resolution, moral or meaning, characteristics of persons involved) to facilitate comparisons across narratives. We jointly analyzed a subset of narratives (n = 30) to identify emerging patterns in the data and develop a codebook, with explicit definitions and examples to ensure coding accuracy and facilitate intercoder reliability.^25^ Three research assistants independently coded the remaining narratives (n = 285), with each member coding an unique sample (n = 84) as well as a shared sample (n = 33) to assess intercoder reliability. All coding discrepancies were resolved by group consensus. We used NVivo 11^26^ for all data management and coding. Our intercoder reliability using the κ coefficient^27^ revealed acceptable agreement among coders (mean [range] κ, 0.93 [0.76-1.00]). We organized codes into themes and subcoded further to characterize dimensions of themes by participant attributes and level of interaction (individual and/or interpersonal, group, and system).

## Results

Of the 315 completed narratives submitted, 3 (1.0%) were from health care system executives, 113 (35.9%) from staff, 97 (30.8%) from academic faculty, 99 (31.4%) from trainees or students, and 3 (1.0%) from participants who declined to specify their positions. Only 48 participants (15.2%) reported being at the institution for less than 1 year, compared with 91 (28.9%) who reported being at the institution for 1 to 5 years, 64 (20.3%) for 5 to 10 years, and 107 (34%) for more than 10 years. More than 90% of participants provided their demographic information, and 188 (59.7%) self-identified as female, 10 (3.2%) as transgender/queer, 38 (12.1%) as non-Hispanic black, and 152 (48.3%) as non-Christian. Also, 31 (9.8%) reported a primary language other than English and 14 (4.4%) reported having a disability (Table 1).

**Table 1.** Respondent Characteristics

Characteristic	No. (%)
Gender identity	
Male	108 (34.3)
Female	188 (59.7)
Transgender/queer^a^	10 (3.2)
Declined to answer^b^	9 (2.9)
Sexual orientation	
Heterosexual	238 (75.6)
Lesbian/gay/homosexual/bisexual^c^	49 (15.6)
Decline to answer	28 (8.9)
Race/ethnicity	
Non-Hispanic white	159 (50.5)
Asian	47 (14.9)
Non-Hispanic black	38 (12.1)
Other^d^	28 (8.9)
Hispanic	21 (6.7)
Multi	11 (3.5)
Declined to answer	11 (3.5)
Belief system	
Non-Christian^e^	152 (48.3)
Christian	151 (47.9)
Declined to answer	12 (3.8)
Primary language	
English	275 (87.3)
Non-English	31 (9.8)
Declined to answer	9 (2.9)
Disability	
Yes	14 (4.4)
No	269 (85.4)
Declined to answer	32 (10.2)
Length of time at institution, y	
<1	48 (15.2)
1-5	91 (28.9)
5-10	64 (20.3)
≥10	107 (34.0)
Declined to answer	5 (1.6)
Position	
Staff^f^	113 (35.9)
Faculty or physician	97 (30.8)
Trainee or graduate student^g^	86 (27.3)
Undergraduate student	13 (4.1)
Executive	3 (1.0)
Declined to answer	3 (1.0)
Primary site	
School of medicine	126 (40.0)
University-affiliated hospitals^h^	103 (32.7)
Free-standing pediatric hospital	21 (6.7)
School of nursing	15 (4.8)
Dental school	13 (4.1)
Outpatient clinics and facilities	9 (2.9)
Research facilities	6 (1.9)
School of social policy and practice	2 (0.6)
Administrative sites	2 (0.6)
Other	11 (3.5)
Declined to answer	7 (2.2)

^a^ Includes “Transgender,” “Other,” and “Do not identify.”

^b^ Declined to answer includes both refused and/or missing responses.

^c^ Includes “Other.”

^d^ Includes Native American/American Indian, Pacific Islander, and “Other (unspecified).”

^e^ Includes all other religious categories.

^f^ Includes “Staff” and “Staff–Manager Level.”

^g^ Includes “Resident/Fellow/Intern/Postdoc” and “Graduate Student.”

^h^ Includes the Veterans Affairs hospital and all other adult care hospitals.

### Narrative Structure

The submitted narratives varied in content but were similar in structure. Most responses detailed a presenting event, reactions, and conclusions. The median (interquartile range) character count of narratives submitted in response to stimulus question 1 was 377 (600); for question 2 it was 66 (90). Despite an online process with assurances of anonymity, some responded that they feared sharing their story, while others often sandwiched their negative experiences with positive statements. Both positive and negative examples of inclusion and lack of inclusion supported the themes we present in the following sections. We aimed to provide quotes most representative of the theme, irrespective of valence.

### Theme 1: A Taxonomy for Characterizing Inclusion

Six broad factors emerged from our analysis to form the basis of a taxonomy that characterizes perceptions of inclusion or lack thereof within health care systems: (1) presence of discrimination; (2) silent witness; (3) interplay among hierarchy, recognition, and civility; (4) effectiveness of leadership and mentors; (5) support for work-life balance; and (6) perceptions of exclusion from inclusion efforts. Regardless of the factor, the underlying thread among these 6 factors was the need to belong and feel recognized and valued. Table 2 summarizes the key factors of an inclusive environment. These factors are also detailed in the following paragraphs with representative quotes.

**Table 2.** Taxonomy of Factors That Foster Inclusion

Key Factors	Representative Quotes
The presence of discrimination	“There are some examples but they are subtle yet apparent. One thing I have noticed is that my residents of color seem to get criticized for things that the majority do not, even if they do the same things. There is this microscope that is applied to them (and I feel myself) which again, is subtle, yet present.”
	“[This institution] like many institutions also has a culture of nepotism and favoritism in the hiring process, which also fosters exclusion. I've had fellow white colleagues nonchalantly mention they were ‘approached’ for promotions. As a woman of color, I've never been ‘approached.’ I’ve had to ‘request’ and self-promote my requests for additional compensation and equal recognition.”
The silent witness	“Though my [department leader] was present for the comment [an insensitive statement relating to sexuality], nothing was said to address the comment that had been made.”
	“I have witnessed a few instances where women or nonwhite students or employees were treated in a disrespectful or discriminatory manner, and I conclude that, despite the rules and policies, it all depends on the particular individuals you end up dealing with.”
The interplay between hierarchy, recognition, and civility	“I sometimes feel that administration sticks to themselves and only interacts with those they view as important people, other professors, or guest speakers. It meant a lot to me that a stranger of a high ranking within the school took the time to get to know someone new who was obviously a trainee, and not directly linked to them or introduced to them.”
	“The only issue I have always had is the feeling of entitlement and rudeness from staff in higher positions that are absolutely rude to staff. When staff members greet certain people they walk by as if nothing was said or just issues with general courtesy—not holding a door when someone is walking in behind you or not holding an elevator door.”
The effectiveness of leadership and mentors	“I have experienced and witnessed leadership in our division systematically do this across ethnic lines, where minorities and outsiders have to work much harder to prove themselves and be treated with respect.”
	“[A mentor] really helped me feel at home here…and gave me an assurance that it would be possible to be able to finish my first year here successfully (which I did, with the help of him and other mentors).”
Support for work-life balance	“[A male colleague] stated that I was not included as an author on a paper to which I contributed before my leave because I ‘made it clear that I was not available to work during my [maternal] leave.’ I have never felt so devalued.”
	“Now having a family of my own, I have asked for time off during my own religious holidays. This has been met with polite ignorance; an unconscious devaluation of non–Judeo-Christian traditions. So I stopped asking.”
Perceptions of exclusion by inclusion efforts	“I’m guessing the inclusion you are speaking of has to do with LGBTQ sorts of issues. I would also imagine that [our institution] does a decent job with this. No firsthand experience.”
	“My one concern about [this institution’s] climate of inclusion is that often, racial and ethnic groups on campus tend to stick together. Unfortunately, these communities often separate themselves from the rest of campus, which creates a conflict between ‘us’ and ‘them’ sometimes.”

#### Presence of Discrimination

Reported discrimination cut across all demographic characteristics and ranged from harassment and bullying to nepotism. Two commonly cited manifestations included microaggressions, defined as casual degradations of any marginalized group, and unequal performance expectations, with males and nonminority groups, often referred to as “the old white boys club,” reportedly benefiting from this inequality. Minorities and women consistently reported being held to stricter standards and needing to work harder to advance within the organization.

#### Silent Witness

Many narratives were submitted by witnesses to discrimination. Witness narratives displayed the impact of discrimination on all parties involved, including fostering anxiety and hindering job performance. Most bystanders disclosed worrying about their own job security and well-being. As one stated, “Some of us whispered about how the [program leader] has done it to other people of color. ‘But do you know how powerful s/he is at [this institution].’ We learned to be silent.”

#### Effectiveness of Organizational Leadership and Mentors

The integral role of leadership and mentorship in fostering discriminatory practices vs promoting inclusion was a common theme. A common perception was that “leadership, the faculty, all hold on to positions of power and promote and protect their own.” Many lamented the lack of mentorship, stating, “people hire and mentor those that have similar demographics.”

#### Interplay of Hierarchy, Recognition, and Civility

Respondents reported differences in treatment and their perceived value based on their status within the organization. Examples of differential treatment varied in degree, from not holding a door or being greeted to perceptions of inadequate compensation, evaluation, or promotion. Narratives characterized status in many ways, including type of position (eg, faculty vs staff, tenure vs nontenure, clinician vs clinician investigator, specialist vs generalist, postdoctoral student vs principal investigator), level of education, gender, and seniority and/or rank.

#### Support for Work-Life Balance

Respondents highlighted how unwritten rules often overshadowed policies put in place to support employees and faculty. Many reported inappropriate comments and/or devaluation of their work on returning from maternity leave or leave for medical reasons. Others praised the supportive actions of their supervisors as examples of an inclusive culture. As one wrote, “He went above and beyond including dedicating space in our center for lactation as well as flexibility to work from home when needed.”

#### Perceptions of Exclusion From Inclusion Efforts

There were many who reported feeling excluded specifically because they were not female or a minority and many others who assumed asking about inclusion must apply only to select groups. Comments ranged from narrow interpretations of inclusion, such as “I suppose, what you mean is in terms of the race and demographics. Then, my comments would not be what your office is seeking” to sentiments such as, “Oh to be a black female and gay! Being none of those things, not only have I been excluded, but I have been intimately involved in processes that required excluding others who are not those things.”

### Theme 2: Inclusion Is Integral to Workforce Wellness and Engagement

Respondents noted how the lack of inclusion they experienced or witnessed affected their well-being and caused stress, anxiety, and feelings of hopelessness, social isolation, and expendability. As one bystander expressed about the department’s approach to an injured colleague, “It left me worried about how I would be treated if I were disabled.” Table 3 further summarizes the effects of noninclusive workplace culture with illustrative quotes.

**Table 3.** The Effects of Organizational Culture on Workforce Wellness and Engagement

Key Theme	Representative Quotes
Fear of repercussions	“The culture of intimidation is such that individual physicians and staff will not report incidents for fear of reprisal and jeopardizing their careers and the expectation that nothing will change.”
Feelings of hopelessness	“Things like this survey will not help because the people in charge of them don't actually have any power to change the climate.”
Feeling expendable	“Only research is valued and respected. Those of us who teach full time are seen as dispensable and not deserving in decision-making processes, including curriculum.”
	“Not only is there little interest on the part of the [principal investigators] to mentor their trainees, but they keep their interactions to a minimum so that it does not feel as though one is contributing—often extremely long hours—to advancing knowledge and the standing of the [principal investigator’s] lab. They may as well have robots doing the work.”
Feelings of social isolation	“Given the incredibly low number of URM students [at this organization], I feel like we stick out like sore thumbs…I don’t feel welcomed here...I felt the pressure to be ‘whiter’ and not allowed to feel comfortable in my own skin/language/culture. I do not feel included.”
	“Unfortunately, this is an experience of exclusion. Over the last several years, within my research group, I have experienced routine isolation and regular condescension, and periodically I have observed my ideas to be ignored until other individuals suggest those ideas.”
Anxiety, stress, depression	“[Shares examples of hyper critical environment that involve public bashing of employees’ errors] First of all, it made me extremely fearful and anxious in my position of making even the most minor mistake and also, what will be said of me by others when I was out of earshot. In all of my years of working, I have never been part of an environment where everyone seems to be so fearful of making mistakes. I’m sad that has been my experience.”
	“Over time, the once bubbly assistant became (what seemed to be) clinically depressed, and so fearful of the director that she literally froze in place whenever she passed by her cubicle. After looking for nearly a year, she secured a new position at [the University], and left the department […] She is currently being treated for PTSD from the experience, while in her new position.”
Lack of engagement	“[After receiving two unsolicited negative comments from a male colleague and a faculty member] Feeling very attacked and degraded by the conversation, I disengaged.”
	“I feel like my career would not prosper [here] due to microaggressions leading to an inability to have my work recognized and my career promoted appropriately. I do not intend to stay at [this institution] after my residency training for these reasons.”

Narratives often described how microaggressions and favoritism eroded participants’ sense of value and thereby limited their engagement and contributions to the organization. As one narrator noted, “Needless to say, I felt exceptionally excluded and no longer want to be engaged in [this health] system. If this is how they treat new people, then it’s not worth my time investing my energy and ideas into the system. I felt very unwelcomed and it still bothers me to this day.”

### Theme 3: Barriers to Challenging Workplace Culture

A common theme that emerged was the lack of effectiveness of formal channels, such as going to human resources, leadership, supervisors, or an ombudsman, to address challenges with workplace culture and interactions. As one witness noted, “The [individual] reported the bullying to HR as well as his/her director’s immediate boss—to no avail. S/He was told s/he was being ‘too sensitive’ when his/her boss made comments about his/her weight.”

Many attempts to confront microaggressions proved futile, as one related, “If I raise a concern [about inappropriate racial/ethnic or sexist comments], they [colleagues] say something sarcastic like ‘sorry I’m not being [politically correct],’ or ‘humor is our way of coping with having to work with very sick people every day.’” Many described self-accommodating behaviors, such as, “my other friends in the office invited [the individual who makes insensitive jokes about ethnicity/race] to lunch, so I started wearing noise canceling headphones and eating alone.” In addition, others referenced resignation and/or transferring as the only solution to their experiences within work environments that lacked inclusion. As one wrote, “Using the words ‘unwelcome’ and ‘devalued’ to describe how I felt [when being pushed off a team after taking family leave] would be understatements. Fortunately, I have found employment elsewhere and will be leaving the [institution’s name] community.”

### Theme 4: Participants’ Recommendations for Fostering Inclusion

Strategies for fostering an inclusive organizational culture emerged from the narratives in the form of direct recommendations and positive examples of inclusion. We found most respondents referenced a systemic culture that influenced their group and interpersonal dynamics. Therefore, recommendations centered on system-level interventions. Table 4 details the respondent-proposed solutions described in the following sections with representative quotes.

**Table 4.** Recommendations From Narratives to Improve Inclusion Within Health Care Organizations

Key Theme	Representative Quotes
**Examine Leadership**	
Leadership and faculty training	“I believe all professors, male and female, should receive unconscious bias training and learn how to interact with diverse students.”
	“I believe mandatory education for faculty on how to accept that they have implicit biases and combat these biases…would be a huge asset to the [institution’s name] community.”
Increase diversity	“Each division or department is different. Mine has explicitly tried to recruit [underrepresented minorities] trainees and faculty. While not [an underrepresented minority] myself, this still creates the kind of environment that I prefer and I think benefits all of us.”
	“Men, and white people, need to recognize that diversity in science and higher ed is ESSENTIAL for them as well.”
**Revisit Organizational Policies**	
Increase accountability	“There are many respectful people at [this institution]. What is lacking is a robust mechanism to appropriately deal with those who are disrespectful.”
Ensure inclusive organizational policies	“One of the reasons for coming to work at [this institution] was the benefit program already in place for domestic partners. I also noticed I didn’t have to hide who I was as a person around my coworkers or boss.”
	“A few months ago, when I received an email offering free yoga and massages to female graduate students only that kind of pissed me off. I definitely felt excluded then.”
**Advocacy Campaigns**	
Promote bystander advocacy	“Older generation attendings do not handle racist/sexist interactions appropriately when they see it—it’s not enough to not be racist/sexist yourself—you have to stand up for other colleagues when patients or coworkers make such comments.”
Promote civility	“However, there are times when exclusion or devaluation is sensed (by me or a colleague) as a result of individuals’ negative disposition and/or heavy tongue/demeanor. It would benefit such individuals as well as those whom they may impact to take a deep breath, lighten up, and ask themselves, ‘what can I do to make someone else’s life better/easier/more pleasant.”
Expand collegial networks	“There is little recognition that perspectives and experiences different than one’s own is what we need to be stronger. This requires a cultivated humility that is somewhat antithetical to the environment.”
	“The environment is generally inclusive, but there is still a tendency of individuals to form insular cliques based on superficial similarities, like shared language. Time will tell if this insularity fades away and opens people to interactions across group regardless of race/ethnic background.”

#### Examining Leadership

Narratives raise the importance of examining leadership, noting, for example, that the institution “needs to educate and strengthen its chairman to change this culture.” Many proposed instituting “mandatory education…on how to accept that they have implicit biases and combat these biases,” starting with leaders and mentors within the organization. Many narratives discussed the importance of diversifying leadership as this quote illustrates: “I am confident we could be a stronger, more resilient and ultimately successful institution if there was authentic engagement of more women at the higher levels of leadership.”

#### Revisit Organizational Policies

Our findings highlighted the strategic need for all health care organizations to examine existing policies, such as family leave or observed religious holidays, to ensure they best meet the needs of a diverse workforce. For example, one respondent noted, “Adoptive parents are not offered any kind of paid parental leave. This makes nontraditional families like mine feel devalued and excluded.”

#### Advocacy Campaigns

A common theme centered on creating a culture and a structure that supports advocacy from those who witness discrimination and/or incivility. Personal narratives routinely stressed the need for bystanders to speak up against discriminatory behaviors rather than remaining silent. For example, one participant stated, “it’s not enough to not be racist/sexist yourself—you have to stand up for other colleagues when patients or coworkers make such comments.” Narratives illustrated the success of bystanders speaking up, as in this example: “I told the patient this [patient requested a student from certain race/ethnicity to leave the room] was unacceptable and either all of us were going to take care of him/her or none of us. It was [his/her] choice. [Patient] apologized and moved on.”

#### Expand Collegial Networks

Respondents emphasized the importance of interacting with different individuals within the organization to gain new perspectives and foster a sense of community by expanding collegial networks. Many respondents, irrespective of position, described the benefits of mentorship on their confidence and ability to manage stressful situations. Some suggested “having mentors and support groups where they discuss interpersonal issues and how to adapt” and proposed pairing employees with mentors who may serve as advocates, role models, and resources when concerns arise.

### Secondary Analyses

When comparing participants grouped by a single attribute, such as race/ethnicity, themes were strikingly consistent across all comparisons. We found minimal differences by position, except for executive leadership, who were underrepresented in our sample. Participants at the institution for less than a year related more positive instances of feeling recognized and fewer events of discrimination, as compared with those who were there longer.

## Discussion

Evaluating and addressing inclusion within health care environments is a new and evolving field. The diversity engagement survey, endorsed by the Association of American Medical Colleges, aims to capture the aspects of institutional culture and social dynamics that sustain an inclusive culture and support the retention of a diverse workforce.^22^ Our narrative analysis augments this survey’s findings^22^ with a deeper understanding and taxonomy of what contributes to an inclusive culture within health care organizations.

Our analysis identified 6 concrete contributors to an inclusive culture that guide tangible strategies to improve inclusivity. This taxonomy is consistent with prior investigations. There is evidence that discrimination manifesting most commonly as unequal expectations and microaggressions for both women^28,29,30,31,32,33^ and minorities^34,35,36^ in medical fields leads to social isolation, disengagement, and burnout not just for those who experience discrimination but for bystanders as well.^37,38^ We see similar findings in other marginalized populations, including non–US-born international medical graduates^39^ or health care professionals and workers with disabilities.^40^ Prior studies also reveal that despite policies in place, persistent challenges in work-life balance hinder female faculty advancement and sense of value within the organization.^41,42,43^ However, our narrative approach was able to provide a nuanced understanding of the concept of inclusion and ways that institutional policies fail to adequately address the problems they attempt to mitigate.

### Is Inclusion a Zero-sum Game?

Inclusion is defined as the “the action or state of including or of being included within a group or structure.” There is no mention of the act of including some at the expense of excluding others. However, our analysis revealed that some perceived that inclusion efforts threaten gains for those who have traditionally thrived in the organization. This sixth factor in our proposed taxonomy for inclusion aligns with prior work. The word *inclusion* appeared to invoke the same perceptions of exclusion or reverse discrimination among the majority culture seen in other efforts to promote diversity.^44,45,46^ For health care organizations and affiliated schools to move forward with inclusion efforts, we need ongoing research on how to design programs that affirm the benefits for all members while providing education to dispel the notion that such efforts are zero-sum.^47,48^

### Implications for the Health Care Workforce

We found that a lack of inclusive culture within health care organizations relates to job performance and emotional wellness. With ongoing efforts to address the wellness of our health care workforce,^49,50,51^ it is important to understand this interplay between an inclusive environment and the wellness of employees, trainees, and students. How we treat each other and how we allow patients to treat us is as important as how we treat patients. Our wellness efforts should reflect this understanding and consider how policy adjustments and training in topics such as cultural humility and implicit biases may assist health care employees, faculty, and students with how they interact with each other^52,53^ as well as address situations where patients may make discriminatory remarks.^54,55^

### Understanding Inclusion

Achieving inclusion starts with effective ways of understanding what predicts it within our health care learning and work environments. Building on surveys that assess culture within health care institutions,^22,56,57^ our targeted online narrative analysis of solicited responses from employees and students provides an effective and innovative method of conducting an audit of organizational inclusion. The need to preserve anonymity and individual voice, avoid social desirability bias, and ensure impunity for members of the workforce and student body makes qualitative assessments using focus groups or semistructured interviews challenging.^58,59,60^ Our study demonstrates that targeted online narrative analysis overcomes the challenges seen with other qualitative methods and provides an anonymous and effective method for conducting ongoing assessments of organizational inclusion. Ongoing assessments of inclusion allow for health care organizations to adapt to evolving workplace cultures.

### Limitations and Strengths

Our study has some limitations. Our findings from a regionally limited set of hospitals and health science schools may not be nationally generalizable. However, prior work in other disciplines supports our results, suggesting that our taxonomy for an inclusive organizational culture may be widely applicable. Our email call for narratives sent out by administrators to their constituencies may be subject to selection bias. We know that executive leadership as well as individuals who self-identified as heteronormative Christian non-Hispanic white males were underrepresented in our sample. However, this narrative analysis by design aims to intentionally capture meaningful qualitative data from individuals within organizations motivated to share their stories.^61^

This study also has some notable strengths. While we reached thematic saturation with a substantially smaller sample size, we still analyzed all completed narratives.^62^ This qualitative assessment of more than 300 stories of inclusion within health care organizations captures experiences from health care professionals, staff, administrators, students, and trainees.

### Achieving Inclusion

Addressing organizational culture is an emerging science in medicine, and we can learn from other disciplines about how to design system-level interventions to achieve inclusion.^63,64^ Consistent with prior work in education, our findings support interventions that expand collegial networks to foster a sense of belonging and community, especially among women and minorities.^65^ Our findings echo the importance of effective leaders and mentors.^66,67^ In addition to diversifying leadership, existing administrators, leaders, and mentors should receive implicit bias training and inclusive leadership skills that include how to be reflective and responsive to feedback.^68,69,70^ Leaders should foster general civility, encourage everyone to speak up against discriminatory acts, and promote policies that advocate for all members of their organization.^66^ A key factor in creating and sustaining an inclusive environment is to empower bystanders and victims alike to speak up against acts of discrimination or incivility.^71,72^ A system for accountability must couple such efforts with policies that support individuals subject to discrimination. Lastly, all health care professionals should possess a working understanding of how unconscious biases may influence daily interactions with colleagues and patients.^73^

## Conclusions

Growing evidence reveals a complex and delicate interplay among how health care professionals treat each other, the wellness and engagement of a diverse workforce, and the care provided to all patients irrespective of their cultural background or personal characteristics.^8,68^ This study provides health care organizations with a novel and effective method for assessing inclusion within health care organizations along with a set of key factors to guide their efforts to operationalize inclusivity. Moreover, our findings underscore the implications of inclusion on wellness and retention of the health care workforce and student body. A focus on factors that promote retention and advancement of a diverse workforce only enhances recruitment efforts of groups underrepresented currently in our workforce.^74^ How we approach both evaluating and addressing inclusion within health care learning and work environments will shape the complex dynamics between the diversity of our health care workforce, the wellness of that workforce, and the care we provide to diverse patient populations.
